# Vapor-Deposited Reactive Coating with Chemically and Topographically Erasable Properties

**DOI:** 10.3390/polym11101595

**Published:** 2019-09-29

**Authors:** Yu-Chih Chiang, Cuei-Ping Ho, Yin-Lin Wang, Po-Chun Chen, Peng-Yuan Wang, Hsien-Yeh Chen

**Affiliations:** 1 School of Dentistry, Graduate Institute of Clinical Dentistry, National Taiwan University and National Taiwan University Hospital, Taipei 10048, Taiwan; 2 Department of Chemical Engineering, National Taiwan University, Taipei 10617, Taiwan; 3 Institute of Materials Science and Engineering, National Taipei University of Technology, Taipei 10608, Taiwan; cpc@mail.ntut.edu.tw; 4 Center for Human Tissues and Organs Degeneration, Institute of Biomedicine and Biotechnology, Shenzhen Institutes of Advanced Technology, Chinese Academy of Sciences, Shenzhen 518055, China; 5 Department of Chemistry and Biotechnology, Swinburne University of Technology, Victoria 3122, Australia; 6 Advanced Research Center for Green Materials Science and Technology, National Taiwan University, Taipei 10617, Taiwan

**Keywords:** poly-*p*-xylylene, coating, surface modification, erasable property, biointerface

## Abstract

An erasable coating was prepared to modify material surfaces with accessibilities, including specific conjugation, elimination of the conjugated chemistry/function, and the reactivation of a second new chemistry/function. The coating was realized based on a vapor-deposited functional poly-*p*-xylylene coating composed of an integrated 3-((3-methylamido)-disulfanyl)propanoic acid functional group, resulting in not only chemical reactivity, but also a disulfide interchange mechanism. Mechanically, the coating was robust in terms of the thermal stability and adhesive property on a variety of substrate materials. Chemically, the anchoring site of carboxylic acid was accessible for specific conjugation, and a disulfide bridge moiety was used to disengage already installed functions/properties. In addition, the homogeneous nature of the vapor-phased coating technique is known for its morphology/thickness and distribution of the functional moiety, which allowed precision to address the installation or erasure of functions and properties. Characterization of the precisely confined hydrophilic/hydrophobic wetting property and the alternating reversibility of this wetting property on the same surface was achieved.

## 1. Introduction

Coating technologies are widely adopted as robust surface modification tools for application on substrate materials to achieve new surface properties. For instance, dopamine-based coatings have been used to create versatile modifications, which were interesting for membranes, particles, and hierarchical materials [1,2,3]. Examples of exploiting chitin, chitosan, and other bio-inspired coatings, have also been shown in various biomedical applications, such as tissue engineering, drug delivery systems, wound healing, cancer treatment, and biosensor design [4,5]. On the other hand, vapor-phase initiators have been deposited on substrates to induce protein adsorption and external stimuli [6,7], among others. Such modification technologies have continued to develop with advanced capabilities, including the following: (i) customizable; (ii) reversible; (iii) dynamic; (iv) precise confinement; and (v) timed control of the surface properties [8,9,10,11]. In this study, we introduce a reactive coating of disulfanyl propanoic acid-functionalized poly-*p*-xylylene (hereafter referred to as an erasable coating), which was prepared based on vapor-phase deposition and a polymerization process. The erasable coating includes a backbone structure of poly-*p*-xylylene, which is analogous to the commercial *Parylene^TM^ N*, *C*, or *D*, but differs in that the side chain has a 3-((3-methylamido)-disulfanyl)propanoic acid substitution [12]. With verified merits of using the functional side chain, the rationale of the coating provides (i) an anchoring site for carboxylic acid that is designed with accessibility for specific conjugation; (ii) a disulfide bridge moiety of the load-lock mechanism [13,14] to disengage already installed functions/properties, which is comparably superior to existing techniques; (iii) a homogeneous coating with a specific coating morphology/thickness and distribution of the functional moiety, which allows precision to address the installation or erasure of functions and properties; and (iv) a coating that is mechanically stable and is applicable for a wide spectrum of substrate materials, topologies, and geometries. The concept of the coating technology enabled location-specific surface modification, i.e., first, a molecule was conjugated at a defined location; then, an erasing procedure was programmed to remove the first conjugated molecule with a defined location at selected areas; and finally, an option to reactivate the surface property via a second conjugation and divergent molecule was manageable during the programmed process. This coating technology results in a state-of-art surface modification platform in which a surface property can have a precisely defined chemical functionality at a confined geographical location, and this definition is erasable and/or reproducible with a new interface property. This surface modification is analogous to the process of illustrating-erasing on a piece of paper.

## 2. Materials and Methods 

### 2.1. Synthesis and Chemical Vapor Deposition (CVD) Polymerization

The synthesis of the starting material of 4-(3-((3-methylamido) disulfanyl)-propanoic acid) [2,2]paracyclophane (dimer) was performed prior to the CVD polymerization to produce the erasable coating. The dimer was synthesized and modified from a commercial compound, [2,2]paracyclophane (Galxyl N, Galentis, Italy), via a five-step route [12]. Briefly, the commercial [2,2]paracyclophane was used as received and was first reacted with 44 mmol a,a-dichloromethyl methyl ether and 77 mmol titanium(IV) chloride for 6 h to form 4-formyl[2,2]paracyclophane. Then, the product was dissolved in a mixture of MeOH and anhydrous tetrahydrofuran and reduced with 28 mmol sodium borohydride for 3 h to yield 4-hydroxymethyl[2,2]paracyclophane. Subsequently, the product was dissolved in anhydrous CH_2_Cl_2_ and reacted with 31.8 mmol PBr_3_ for 4 h to produce 4-bromomethyl[2,2]paracyclophane. Then, 80% aqueous hydrazine was added to yield 4-aminomethyl[2,2]paracyclophane. Next, the 4-aminomethyl[2,2]paracyclophane was reacted with 10 mmol 3,3′- dithiodipropionic acid and 10 mmol N- ethyl-N′-(3-(dimethylamino)propyl)carbodiimide in anhydrous tetrahydrofuran for 20 min, resulting in the final product of 4-(3-((3-methylamido)disulfanyl)propanoic acid) [2,2]paracyclophane. Upon obtaining the final dimer product, preparation of the erasable coating was thus performed via the CVD polymerization process of this dimer. During the CVD process, 4-(3-((3-methylamido) disulfanyl)-propanoic acid) [2,2]paracyclophane was first sublimated at 125 °C, and the vaporized dimers were then subjected to a pyrolysis temperature of 550 °C to turn the dimers into reactive monomers (*p*-quinodimethane). Finally, the monomers were transferred to a deposition chamber and polymerized upon condensation/deposition at 20 °C to form the erasable coating. A reduced pressure of 100 mTorr was maintained throughout the entire CVD process, and the deposition rate was regulated and monitored by a thin-film monitor (STM-100/MF, Sycon Instruments, East Syracuse, NY, USA) at approximately 0.5 to 1.0 A°/s.

### 2.2. Immobilization

Fluorescein-labeled Arg-Arg-Cys-Cys peptide (Yao-Hong Biotechnology Inc., Taiwan) was reacted with the coating surface by the addition of 5 mM 1-ethyl-3-(3-dimethylaminopropyl)-carbodiimide (Alfa Aesar, Haverhill, MA, USA) with 5 mM N-hydroxysuccinimide (Alfa Aesar, Haverhill, MA, USA) for 2 h at 4 °C, and a microcontact printing (μCP) process [15,16] was used to localize the reaction in selected areas. The resulting samples were washed with phosphate-buffered saline (PBS, contains Tween 20, Sigma-Aldrich, St. Louis, MO, USA) three times and with deionized water twice to remove excess and unreacted reagents. The erase process was performed by using a 100 mM glutathione (Sigma-Aldrich, St. Louis, MO, USA) reductant to treat the samples for 6 h at 20 °C, and a similar μCP process (oval pattern, major axis = 50 μm, minor axis = 20 μm) was again used to localize the reaction to selected areas. A similar wash process using PBS three times and deionized water twice was used after the reaction to remove excess and unreacted reagents. The reactivation process was demonstrated by reacting 100 mM cysteamine (Sigma-Aldrich, St. Louis, MO, USA) with the sample surface via the addition of 1 mM 2,2’-dithiodipyridine (DTP; Sigma- Aldrich, St. Louis, MO, USA) for 6 h at 4 °C. Finally, a 10 mM Alexa Fluor 350 NHS ester (Life Technologies, Carlsbad, CA, USA) was used to confirm that conjugated cysteamine was on the coating surface. A fluorescence microscope (TE2000-U, Nikon, Tokyo, Japan) was used to examine the modified sample surfaces. However, to demonstrate the hydrophobic/hydrophilic modification of the erasable coating, the hydrophilic molecule of cysteamine (100 mM) and the hydrophobic perfluorodecanethiol (5 vol% in ethanol, Sigma-Aldrich, St. Louis, MO, USA) were used for the same conjugation and erasing process mentioned above. The same wash process was also conducted during the hydrophobic/hydrophilic modification experiments.

### 2.3. Surface Characterization

FT-IR spectra were recorded with a Spectrum 100 FT-IR spectrometer (PerkinElmer, Waltham, MA, USA). A liquid nitrogen-cooled mercury-cadmium-telluride (MCT) detector and an advanced grazing angle specular reflectance accessory (PIKE Technologies, Fitchburg, WI, USA) were used during the spectra acquisition. The scan range was from 500 cm^−1^ to 4000 cm^–1^, and 64 scans were performed for each acquisition. For the coating adhesion experiments, the coated samples were purposely scratched with a metal multi-blade (ZCC 2087 cross-cut tester, Zehntner, Sissach, Switzerland). The scratched samples were then applied with Scotch^TM^ adhesive tape (3M, Maplewood, MN, USA) and were subsequently subjected to a quick-removal process to remove the Scotch^TM^ tape from the sample surfaces. The resulting samples were examined with a scanning electron microscope (SEM, Nova NanoSEM 230, FEI, Hillsboro, OR, USA), which was operated under a reduced pressure of 4 × 10^–6^ Torr. During the SEM acquisition, the acceleration voltage was 10.0 kV and the working distance was approximately 5.4 mm. Elemental maps were recorded and analysed with the energy dispersive X-ray analysis (EDX) feature of the same SEM instrument. The thermal stability of the coating was examined by exposing the same coating samples in a forced air-drying oven (OVP30, Hondwen, Taiwan) under an elevated temperature from 25 °C to 210 °C for 2 h for each temperature increment. The resulting changes in the chemical compositions were analysed and compared with the recorded FT-IR spectra from the same Spectrum 100 FT-IR instrument mentioned above. Surface wetting properties were examined via a water condensation experiment, which was performed by spraying and drying a water film on the modified surfaces, and the sample surface was then visualized with a microscope (Olympus, Tokyo, Japan). The water contact angle was measured at room temperature by using a contact angle goniometer (First Ten Angstroms, Portsmouth, VA, USA) upon the placement of 5 μl of distilled water on the surfaces. Each measurement was conducted using three different locations on the same sample and repeated for different samples in triplicate.

## 3. Results and Discussion

**Figure 1:**
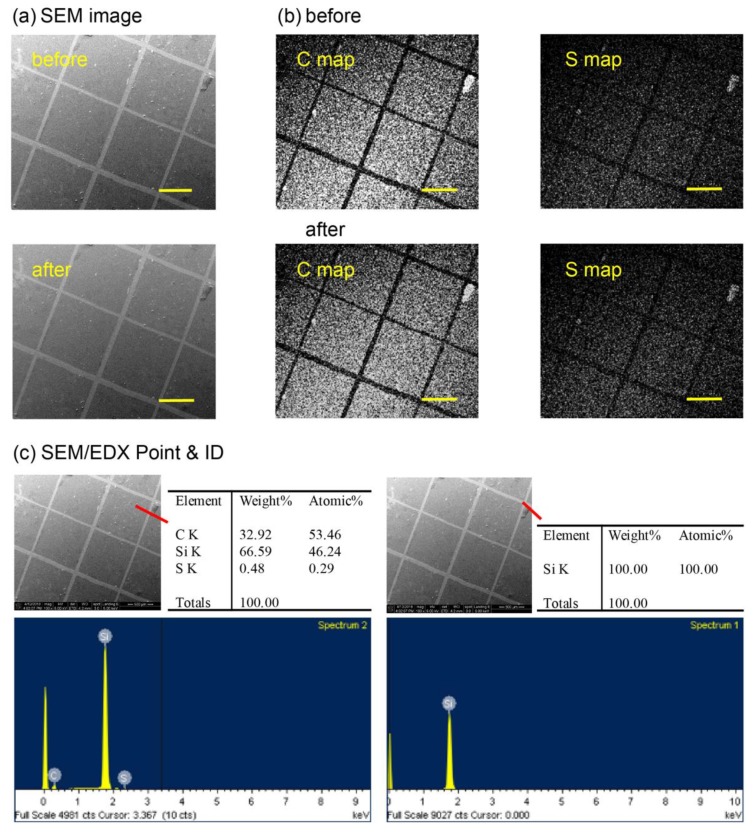
The mechanical adhesion of the erasable coating on the substrates was examined via an adhesion test. (**a**) Scanning electron microscope (SEM) images of the erasable coating before and after the cross-cut tape adhesion test. (**b**) SEM/energy dispersive X-ray analysis (EDX) elemental maps of carbon (C) and sulfur (S) showed no apparent damage after the adhesion test compared with those before the test. (**c**) SEM/EDX Point and ID precisely indicated carbon (C), sulfur (S), and silicon (Si) on the intact regions (square-shaped areas), whereas only Si was detected on the bare substrate. Scale bars: 600 μm.

Preparation of the coating was realized by using a chemical vapor deposition (CVD) polymerization process from the 3-((3-methylamido)-disulfanyl)propanoic acid-substituted [2.2]paracyclophane (dimer) starting material, whose synthesis details are included in the Materials and Methods. During the CVD process, the dimer was vaporized at approximately 125 °C and then subjected to pyrolysis treatment at 550 °C to ensure the transformation into highly reactive *p*-quinodimethanes (monomers). The monomers then underwent radical polymerization to deposit the erasable coating on substrates at approximately 20 °C. The coating thickness was controlled based on the time duration of the CVD process and was from 100 nm to 150 nm, as measured based on the in-situ quartz crystal microbalance (QCM) and also by spectroscopic ellipsometry after retrieving the coated samples from the CVD system. The resulting coatings exhibited excellent mechanical adhesion on the substrates compared to other *Parylene^TM^* and derivatives [17,18], and the adhesion test results agree with the American Society for Testing and Materials (ASTM), with a high standard of class 5B [19]. As revealed in Figure 1, the SEM images and EDX unambiguously confirmed the adhesive properties, and also revealed the coating conformity and the homogeneousness of the coating by confirming the anticipated and consistent chemical composition of 53.46 atom-% carbon (C), 46.24 atom-% silicon (Si), and 0.29 atom-% sulfur (S) at various locations on the same coating surface, whereas 100.00 atom-% silicon (Si) was found for the bare substrate (by harshly removing the coating by a cutting blade). However, the thermal stability of the coating was also evaluated by exposing the coating samples to elevated temperatures of 25 °C, 90 °C, 130 °C, 170 °C, and 210 °C, and the possible compositional changes were examined based on FT-IR analysis. The recorded spectra showed characteristic peaks of the C=O asymmetric stretch at 1662 cm^–1^ and the C–N stretch at 1036 cm^–1^, detected from 25 °C to 170 °C, which verified the existence of a secondary amide side group. Additionally, no signals were observed for these peaks at 210 °C, which may be due to possible decomposition of the side chain. Furthermore, a considerable drop in intensity at 210 °C for the overlapped adsorption bands of the N–H and O–H stretches from 3114 cm^–1^ to 3520 cm^–1^ ambiguously confirmed the instability of the side group at this temperature. Notably, the characteristic adsorption peaks of C–H at 2857, 2923, and 2958 cm^–1^ were detected with decreasing peak intensities with an increase in treatment temperature, and a possible decomposition of the backbone structure of poly-*p*-xylylene occurred at approximately 90 °C. The thermal stability data are included in the Supplementary Materials in Figure S1. In light of the similarity to other functional poly-*p*-xylylene systems using a vapor-based deposition process, which have been demonstrated to be successfully modified on materials including metals, oxides, alloys, polymers, glass, silicon, and liquids [20,21,22], with excellent mechanical stabilities, including corrosion resistance, enhanced lubricity, a high tensile strength, and surface consolidation to avoid flaking or dusting [23], the present coating technique is theoretically applicable to a wide range of materials.

**Figure 2:**
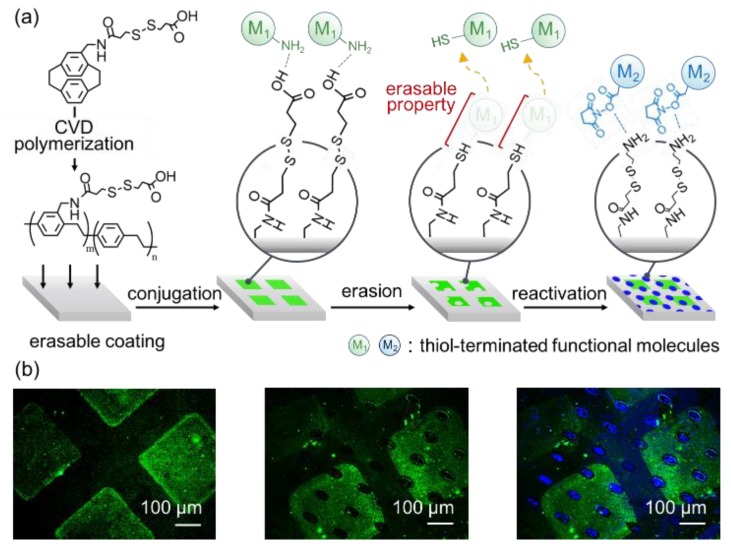
(**a**) Schematic illustration of the surface modification platform based on the erasable coating to provide spatially confined accessibilities of (i) specific conjugation to alter the surface chemistry, (ii) an erasable mechanism to remove the existing chemistry, and (iii) reactivation of the surface property for a second divergent chemistry via a disulfide interchange route. (**b**) A demonstration of using the erasable coating to confine the conjugations of multiple fluorescent molecules on the same surface. Fluorescein (FITC)-Arg-Arg-Cys-Cys (RRCC) (green signals) was conjugated to the surface at selected areas in a 300 μm × 300 μm square pattern (fluorescence micrograph on the left), and subsequent removal of the conjugated FITC-RRCC occurred with a second independent oval pattern (major axis = 50 μm, minor axis = 20 μm) in the middle (erased green signals). Finally, reactivation occurred to install a divergent molecule system of cysteamine/Alexa Fluor 350 NHS ester (blue channel) in the oval pattern areas, as shown to the right.

**Figure 3:**
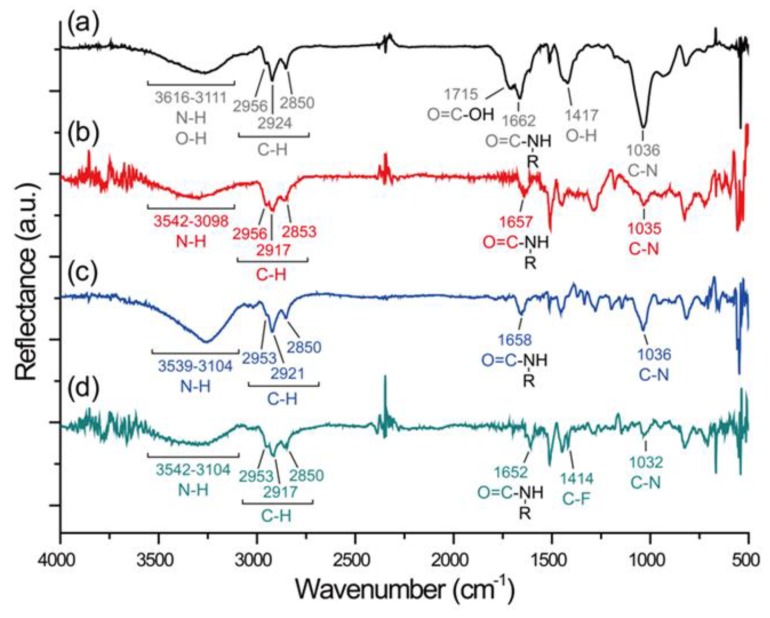
FT-IR spectral characterization used to monitor the surface chemistry modifications for the erasable coating. The FT-IR spectra were recorded to compare (**a**) the as-deposited erasable coating with (**b**) the erased treatment of the coating, (**c**) the conjugation with cysteamine, and (**d**) the conjugation with perfluorodecanethiol. The surface modifications were conducted on the same sample surface.

The important and unique capability of the erasable coating to control interface properties by offering (i) specific conjugation accessibility, (ii) an erasable capability to disengage the already-installed chemistry/property in (i), and (iii) precise confinement of (i) and (ii) in selected areas of interests is illustrated and demonstrated in Figure 2a. The end group of the erasable coating provides abundant carboxyl moieties that are readily accessible via the conjugation reaction with an amino group, forming a peptide bond in the well-known 1-ethyl-3-(3-dimethylaminopropyl)-carbodiimide (EDC) and N-hydroxysuccinimide (NHS) environment [24]. In the experiment, a model fluorescence probe of fluorescein (FITC, green channel)-conjugated Arg-Arg-Cys-Cys (RRCC) peptide, which also contains abundant amino groups, was allowed to react with the coating surface, and control over spatial confinement was enabled by the assistance of a microcontact printing (μCP) technique. The μCP was also used to form a uniform coating and homogeneously distributed functionality on the coating surface, providing an intimate engagement between the two contact surfaces [15,16]. As shown in Figure 2b, the FITC signals were detected in the anticipated areas, displaying good agreement with the pattern (squares, 300 μm × 300 μm) used during the μCP, which indicated successful conjugation and confinement when using the end groups. Subsequently, an erasing process to disengage the immobilized FITC−RRCC was performed via a disulfide interchange reaction under glutathione conditions [25,26,27], which was also applied to the surface via the μCP confinement technique and a divergent pattern (oval pattern, major axis = 50 μm, minor axis = 20 μm) for better demonstration of the concept. The erasure removed the FITC signals at the correct locations (if previously installed) that correspond to the anticipated oval pattern of the μCP stamp. The disulfide interchange reaction of the disulfanyl propanoic acid moiety on the erasable coating provided a more exciting route to reinstall the second chemistry/function after the erasure was performed. As discovered in the experiments, the confined areas of erasure exhibited reactivity towards a thiol-active compound, i.e., cysteamine, and conjugation via a second disulfide interchange reaction efficiently resulted in the immobilization of cysteamine. The detection of cysteamine was confirmed by a previously modified Alexa Fluor 350 NHS ester (blue channel) on the cysteamine, which was found to be well-distributed in the areas of the oval patterns. The resulting modification on the coating surface exhibited (i) elegant manipulation of the immobilization/cleavage of molecules on the surface, enabling installation/erasure of the surface chemistry of interest, and (ii) precise confinement of this manipulation to provide geographical control. Since the area or pattern that already installed the first chemistry/property and the area or pattern that subsequently overwrote the second chemistry/property can be different, the resulting modification surface will be multifunctional (i.e., areas of the first functional molecule, the second functional molecule, and no functional molecules coexist on the surface), with a controllable distribution. Verification of the conjugation chemistry was conducted via FT-IR analysis. The recorded FT-IR spectra of the pure erasable coating (Figure 3a) was first compared to a coating that was treated with an erasing process. A significantly reduced peak intensity for C=O stretches at 1715 cm^–1^ and for the O–H single bond bends at 1417 cm^–1^ showed the decomposition of disulfide bonds after this treatment (Figure 3b). However, the conjugation of cysteamine produced increased intensities in the range of 3111 cm^–1^ to 3616 cm^–1^, which indicated amine adsorption from the conjugated cysteamine (Figure 3c).

**Figure 4:**
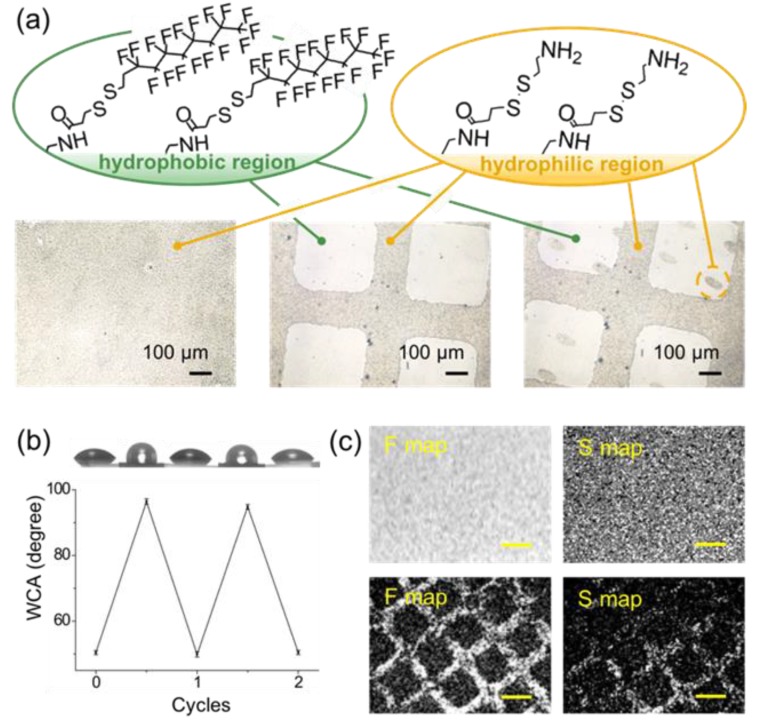
Confinement and manipulation of the surface wetting properties. (**a**) A hydrophilic molecule of cysteamine was conjugated to the erasable coating, creating a homogeneous hydrophilic surface with well-adsorbed water molecules, as shown in the left micrograph. The same erasing process was performed to selectively remove the conjugated cysteamine to result in a confined area with a low adsorption of water molecules (hydrophobic), as exhibited in the middle micrograph. A reinstallation of cysteamine onto the same surface and with an independent pattern system was performed to render a hierarchical hydrophobic/hydrophilic property, as shown on the right. (**b**) Statistical analysis of altering the wetting properties during two complete cycles. A recovery rate of 92.6 ± 2.8% was estimated. (**c**) EDX elemental maps of fluorine (F) and sulfur (S) to confirm the conjugation of perfluorodecanethiol on the coating surface. The erasure process used to selectively remove the conjugated perfluorodecanethiol showed reduced signals for F and S, with a consistency in the confinement patterns. Scale bars: 300 μm.

Finally, the important surface wetting properties (hydrophobicity and surface energy) were manipulated by controlling the (i) sophisticated, hybrid presentation of the wetting property at precisely confined locations for the modifications, and the (ii) reversibility of alternating the wetting property, i.e., erasability and/or alteration of the wettability from a hydrophobic state to a hydrophilic state or vice versa. In the demonstration shown in Figure 4a, coating surfaces were uniformly modified with cysteamine, yielding an overall hydrophilic surface; subsequently, the same erasing process was used to remove cysteamine from the selected areas. Additionally, we used the disulfide interchange reaction to conjugate/install a hydrophobic perfluorodecanethiol in the vacated areas. The successful conjugation of perfluorodecanethiol was also verified in a separate experiment, which showed an increased peak adsorption for C–F at 1414 cm^–1^ in the FT-IR analysis, as demonstrated in in Figure 3d. This straightforward erasure and reinstallation process results in a hybrid wetting property that provides both hydrophobic and hydrophilic pockets on the coating surfaces, as shown in the water condensation experiments (allowing water molecules to adsorb onto the surface). Moreover, upon continuing the same erasing and reinstallation process on the same sample surface, a state-of-the-art hierarchical hydrophobic/hydrophilic property was created with elegant control in defined and confined areas on the coating surfaces. A more statistical demonstration was performed by measuring the water contact angles (WCA) of the modified surfaces, which showed consistent alterations between hydrophilic (WCA = 50 degrees) and hydrophobic (WCA = 96 degrees) states during two complete cycles (equal to four erase cycles), as shown in Figure 4b. A recovery rate of 92.6 ± 2.8% was estimated for such a hydrophobic/hydrophilic alteration, and a 88.5 ± 3.7% conversion rate for the erasure process (Supplementary Materials, Figure S2) also unambiguously verified the results. The data were also well-supported by the EDX elemental map analysis based on a comparison before and after the erasure process used to remove the previously installed perfluorodecanethiols from the coating surface. As indicated in Figure 4c, strong signals from fluorine were detected throughout the coating surface, which indicated the homogeneous and successful conjugation/installation of the perfluorodecanethiol. Subsequently, erasure via the disulfide interchange reaction occurred in selected areas and resulted in a significant reduction of the fluorine signals in these areas. However, a detectable reduction in sulfur intensity between the disulfides and the thiols was also found on the coating surface after the erasure and with a good consistency of the mapping pattern compared with the fluorine and the pattern during the erasing process.

## 4. Conclusions

Advanced control over the interfacial properties, including the specific conjugation accessibility, a homogeneous and high molecular resolution to enable precise geographical control, a capability to eliminate existing properties, and a timed flexibility for manipulating the properties, were realized by using the erasable coating introduced in this study. The coating technique exhibited inherent merits of being a vapor-deposited conformal coating, having a long-term stability and a high biocompatibility, compared to its predecessors, the poly-*p*-xylylenes, and the coating process is expected to be applicable to substrates and devices, regardless of the materials or the shape. With the feasibility of exploiting copolymerization with other functional poly-*p*-xylylene derivatives that are already on the shelves, in order to create multifunctional coatings, we foresee uses of the coating technology to create prospective and more sophisticated interfacial properties that are hampered by current technologies.

## Supplementary Materials

The following are available online at https://www.mdpi.com/2073-4360/11/10/1595/s1, Figure S1: FT-IR spectra showing the thermal stability of the coating. Figure S2: Reaction efficiency analysis of the erasable coating. Figure S2: Reaction efficiency analysis of the erasable coating.
