# Supplementary material for: Vapor-Deposited Reactive Coating with Chemically and Topographically Erasable Properties
Source: Polymers (Basel). 2019 Sep 29;11(10):1595. doi: 10.3390/polym11101595 (PMC6835693; doi:10.3390/polym11101595)
Supplement: Supplementary file 1 [file polymers-11-01595-s001.pdf]

Supplementary Materials

Vapour-Deposited Reactive Coating with Chemically and Topographically Erasable Properties

Yu-Chih Chiang [3,†], Cuei-Ping Ho [1,†], Yin-Lin Wang [3], Po-Chun Chen [4], Peng-Yuan Wang [5,6,*] and Hsien-Yeh Chen [1,2,*]

[1] Department of Chemical Engineering, National Taiwan University, Taipei 10617, Taiwan
[2] Advanced Research Center for Green Materials Science and Technology, National Taiwan University, Taipei 10617, Taiwan
[3] School of Dentistry, Graduate Institute of Clinical Dentistry, National Taiwan University and National Taiwan University Hospital, Taipei, 10048, Taiwan
[4] Institute of Materials Science and Engineering, National Taipei University of Technology, Taipei 10608, Taiwan
[5] Center for Human Tissues and Organs Degeneration, Institute of Biomedicine and Biotechnology, Shenzhen Institutes of Advanced Technology, Chinese Academy of Sciences, Shenzhen 518055, China
[6] Department of Chemistry and Biotechnology, Swinburne University of Technology, Victoria 3122, Australia

* Correspondence: cpc@mail.ntut.edu.tw (P-C. C); py.wang@siat.ac.cn (P-Y. W); hsychen@ntu.edu.tw (H-Y. C) Tel. +886-2-33669476; Fax. +886-2-23623040

† Y.-C. Chiang and C.-P. Ho contributed equally to this work.

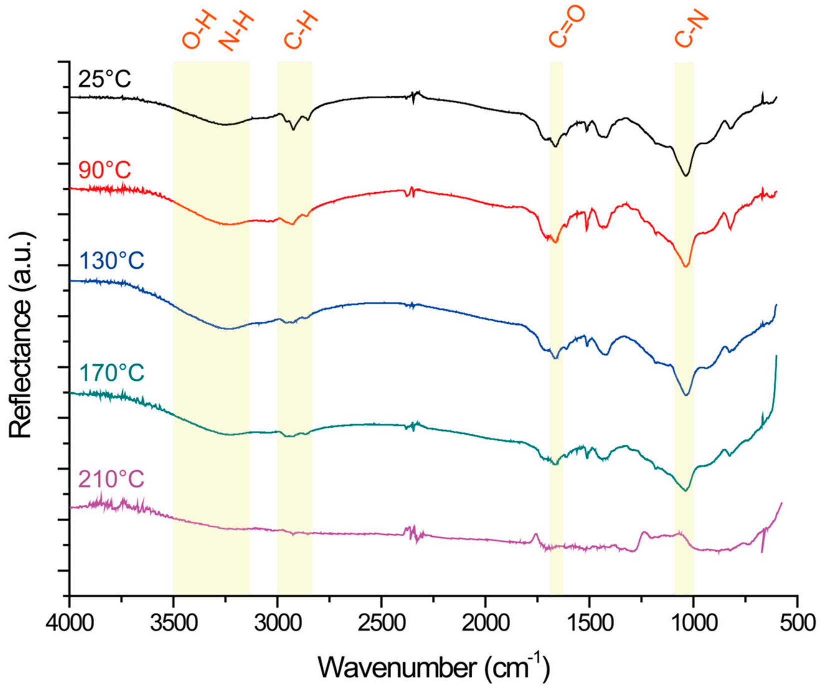

Figure S1. FT-IR spectra showing the thermal stability of the erasable coating at 25 °C, 90 °C, 130 °C, 170 °C and 210 °C. The film remained thermally stable from 25 °C to 170 °C based on negligible changes in the position and intensity of the characteristic bands of *C*=*O*, *C*−*N*, *N*−*H*, and *O*−*H*. However, the decreased intensities for the *C*=*O*, *C*−*N*, *N*−*H*, and *O*−*H* peaks suggested a limitation of the thermal stability at 210 °C.

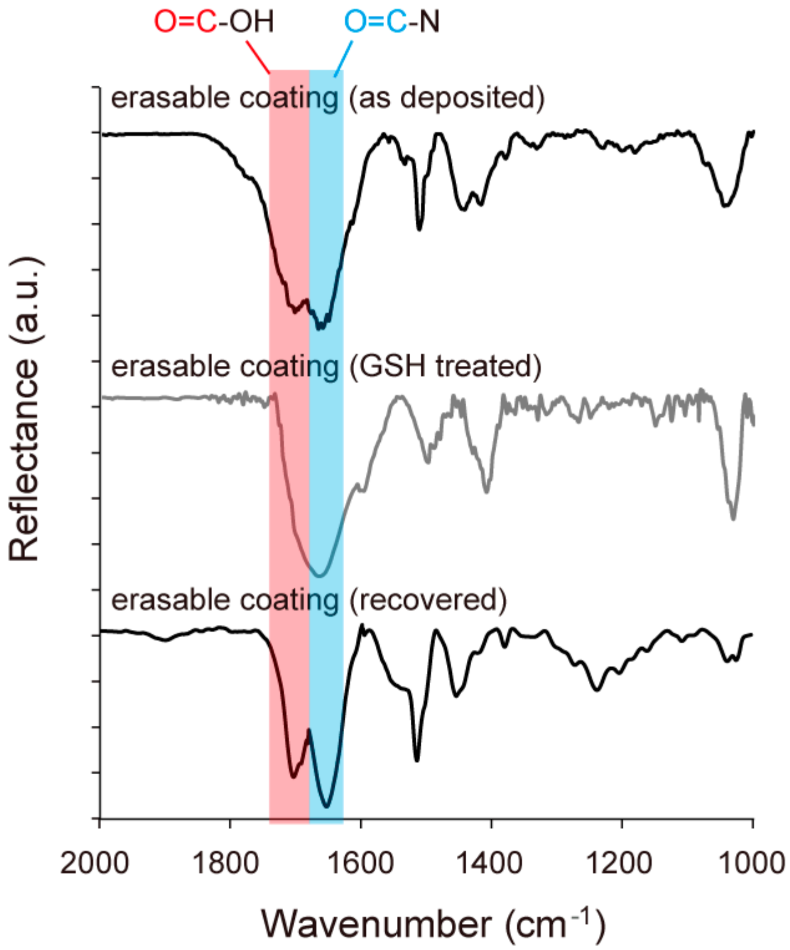

Figure S2. Reaction efficiency analysis of the erasable coating. FT-IR spectra were recorded for the coating before (as deposited) and after modifications including GSH treatment and recovering the coating with 3-mercaptopropionic acid. A selected range of scans from 1000 to 2000 cm^{-1} was used during the FT-IR acquisition. Experiments were performed on the same sample surface, and a high efficiency of 88.5 ± 3.7% was discovered based on comparing the integrated peak areas of *O=C-OH* (with respect to unchanged areas for *O=C-N*).
